# Acid‐sensing ion channel 1a drives AMPA receptor plasticity following ischaemia and acidosis in hippocampal CA1 neurons

**DOI:** 10.1113/JP270701

**Published:** 2015-08-18

**Authors:** Patrice Quintana, David Soto, Olivier Poirot, Marzieh Zonouzi, Stephan Kellenberger, Dominique Muller, Roman Chrast, Stuart G. Cull‐Candy

**Affiliations:** ^1^ Department of Basic NeurosciencesUniversity of Geneva1211 Geneva 4Switzerland; ^2^ Department of Neuroscience, Physiology and PharmacologyUniversity College LondonGower StreetLondonWC1E 6BTUK; ^3^ Molecular Nociception Group, Wolfson Institute for Biomedical ResearchUniversity College LondonLondonWC1E 6BTUK; ^4^ Department of Pharmacology and ToxicologyUniversity of Lausanne1005LausanneSwitzerland; ^5^ Department of Medical GeneticsUniversity of Lausanne1005LausanneSwitzerland

## Abstract

**Key points:**

- The hippocampal CA1 region is highly vulnerable to ischaemic stroke. Two forms of AMPA receptor (AMPAR) plasticity – an anoxic form of long‐term potentiation and a delayed increase in Ca^2+^‐permeable (CP) AMPARs – contribute to this susceptibility by increasing excitotoxicity.
- In CA1, the acid‐sensing ion channel 1a (ASIC1a) is known to facilitate LTP and contribute to ischaemic acidotoxicity.
- We have examined the role of ASIC1a in AMPAR ischaemic plasticity in organotypic hippocampal slice cultures exposed to oxygen glucose deprivation (a model of ischaemic stroke), and in hippocampal pyramidal neuron cultures exposed to acidosis.
- We find that ASIC1a activation promotes both forms of AMPAR plasticity and that neuroprotection, by inhibiting ASIC1a, circumvents any further benefit of blocking CP‐AMPARs.
- Our observations establish a new interaction between acidotoxicity and excitotoxicity, and provide insight into the role of ASIC1a and CP‐AMPARs in neurodegeneration. Specifically, we propose that ASIC1a activation drives certain post‐ischaemic forms of CP‐AMPAR plasticity.

**Abstract:**

The CA1 region of the hippocampus is particularly vulnerable to ischaemic damage. While NMDA receptors play a major role in excitotoxicity, it is thought to be exacerbated in this region by two forms of post‐ischaemic AMPA receptor (AMPAR) plasticity – namely, anoxic long‐term potentiation (a‐LTP), and a delayed increase in the prevalence of Ca^2+^‐permeable GluA2‐lacking AMPARs (CP‐AMPARs). The acid‐sensing ion channel 1a (ASIC1a), which is expressed in CA1 pyramidal neurons, is also known to contribute to post‐ischaemic neuronal death and to physiologically induced LTP. This raises the question does ASIC1a activation drive the post‐ischaemic forms of AMPAR plasticity in CA1 pyramidal neurons? We have tested this by examining organotypic hippocampal slice cultures (OHSCs) exposed to oxygen glucose deprivation (OGD), and dissociated cultures of hippocampal pyramidal neurons (HPNs) exposed to low pH (acidosis). We find that both a‐LTP and the delayed increase in the prevalence of CP‐AMPARs are dependent on ASIC1a activation during ischaemia. Indeed, acidosis alone is sufficient to induce the increase in CP‐AMPARs. We also find that inhibition of ASIC1a channels circumvents any potential neuroprotective benefit arising from block of CP‐AMPARs. By demonstrating that ASIC1a activation contributes to post‐ischaemic AMPAR plasticity, our results identify a functional interaction between acidotoxicity and excitotoxicity in hippocampal CA1 cells, and provide insight into the role of ASIC1a and CP‐AMPARs as potential drug targets for neuroprotection. We thus propose that ASIC1a activation can drive certain forms of CP‐AMPAR plasticity, and that inhibiting ASIC1a affords neuroprotection.

**Abbreviations**
a‐LTP: anoxic LTP
AMPAR: AMPA receptor
ASIC1a: acid‐sensing ion channel 1a
CP‐AMPAR: calcium‐permeable AMPAR
HPN: hippocampal pyramidal neuron
*I–V*: current–voltage
KO: knockout
LTP: long‐term potentiation
NASPM: 1‐naphthyl acetyl spermine
NFATc: nuclear factor of activated T cells
NMDAR: NMDA receptor
OGD: oxygen glucose deprivation
OHSC: organotypic hippocampal slice culture
PcTx1: psalmotoxin 1
PI: propidium iodide
RI: rectification index
TBS: theta‐burst stimulation
WT: wild type

## Introduction

During ischaemia, the excessive release of the excitatory neurotransmitter glutamate leads to neuronal toxicity and death, in part due to over‐activation of ligand gated ion channels and the resultant calcium entry. While early work suggested that excitotoxicity due to excessive activation of NMDA receptors (NMDARs) was the main cause of neuronal death in the ischaemic brain, it has become apparent that the situation is more complex, involving activation or alteration of a host of ionotropic receptors and channels (Szydlowska & Tymianski, 2010), including calcium‐permeable AMPA receptors (CP‐AMPARs) (Pellegrini‐Giampietro *et al*. 1992; Opitz *et al*. 2000; Tanaka *et al*. 2002; Noh *et al*. 2005) and acid‐sensing ion channels (ASICs) activated by the increased protons associated with ischaemia (Xiong *et al*. 2004; Pignataro *et al*. 2007; Mari *et al*. 2010; Sherwood *et al*. 2011). How these different membrane channels contribute and interact to confer ischaemic susceptibility is still poorly understood.

The hippocampal CA1 area is critical for several types of memory and learning (Bartsch *et al*. 2011; Goshen *et al*. 2011). CA1 hippocampal pyramidal neurons (HPNs) are particularly susceptible to ischaemia, which has long been known to trigger their delayed death in humans and animal models (Kirino, 1982; Petito *et al*. 1987). Two forms of post‐ischaemic AMPA receptor plasticity have been suggested to intensify the excitotoxicity that occurs within the CA1 region, and to contribute to the HPN delayed degeneration. First, there is a persistent enhancement in glutamatergic neurotransmission, the anoxic long‐term potentiation (a‐LTP), which occurs within the first hour following ischaemia (Hsu & Huang, 1997; Kawai *et al*. 1998; Quintana *et al*. 2006; Dias *et al*. 2013). Second, there is evidence for a downregulation of GluA2 expression several hours later. This is accompanied by enhanced expression of Ca^2+^‐permeable GluA2‐lacking AMPARs (CP‐AMPARs) (Pellegrini‐Giampietro *et al*. 1992; Opitz *et al*. 2000; Tanaka *et al*. 2002; Noh *et al*. 2005). Pharmacological block of CP‐AMPARs with 1‐naphthyl acetyl spermine (NASPM) has been shown to provide a striking time window of ∼40 h of neuroprotection in the CA1 area (Noh *et al*. 2005), in keeping with the view that these CP‐AMPARs play a critical role in cell death. It is of note that CP‐AMPARs are expressed during normal LTP at CA1 synapses although in these conditions they are present only transiently (Plant *et al*. 2006; Morita *et al*. 2014; but see Adesnik & Nicoll, 2007).

Hippocampal pyramidal neurons in CA1 also express the sodium‐ and calcium‐permeable ASIC1a channels (Wemmie *et al*. 2013). Previous studies have demonstrated that following focal ischaemia, the pH typically falls to 6.5–6.0 under normo‐glycaemic conditions and can fall below 6.0 during severe ischaemia or hyperglycaemia (Siesjo, 1982; Rehncrona, 1985; Nedergaard *et al*. 1991). More recent work has demonstrated the involvement of ASIC1a homomers and ASIC1a/2b heteromers (channels we refer to here as ASIC1a) in the neurotoxicity induced by ischaemic acidosis (acidotoxicity), possibly due to their calcium permeability (Xiong *et al*. 2004; Pignataro *et al*. 2007; Mari *et al*. 2010; Sherwood *et al*. 2011). Thus, ASIC1a blockers or knockout (KO) of the ASIC1a gene provides significant neuroprotection *in vivo* (Xiong *et al*. 2004; Pignataro *et al*. 2007). In CA1 HPNs, this deleterious role of ASIC1a is exacerbated by its functional coupling with NMDA receptors (NMDARs; Gao *et al*. 2005). One of the physiological roles of ASIC1a appears to be regulation of AMPAR synaptic plasticity. Thus, loss of ASIC1a in KO mice impairs activity‐dependent LTP, without modification of basal AMPAR‐mediated neurotransmission both in CA1 cells (Wemmie *et al*. 2002) and in lateral amygdala (Du *et al*. 2014).

Here we have examined the possibility that ASIC1a activation can promote post‐ischaemic AMPAR plasticity in CA1 HPNs, and may play a role in the vulnerability of this region to anoxic injury.

## Methods

### Ethical approval

For hippocampal pyramidal neuron cultures, rats were killed by decapitation in accordance with the UK Animals (Scientific Procedures) Act 1986. For organotypic hippocampal slice cultures, mice were killed by decapitation, using a protocol approved by the Geneva and Vaud Veterinarian Office.

### Organotypic slice cultures and anoxia/hypoglycaemia induction

ASIC1a KO mice were kindly provided by Michael Welsh. The knockout ablates all ASIC1a‐containing channels and is therefore expected to result in loss of calcium‐permeable ASIC1a channel (ASIC1a homomers and ASIC1a/2b heteromers) and the Ca^2+^‐impermeable ASIC1a/2a heteromers. Organotypic hippocampal slice cultures (OHSCs) from 4‐ to 5‐day‐old mouse (P4–5) were prepared and maintained for 10–15 days as described previously (Stoppini *et al*. 1991). Oxygen glucose deprivation (OGD) was induced for 10 min as previously described (Quintana *et al*. 2006). Briefly, slices were placed in a homemade chamber and continuously perfused with artificial cerebrospinal fluid (ACSF) using a peristaltic pump with a flow rate of 2.5–3 ml min^−1^, temperature 35 ± 1°C. Control slices were exposed to ACSF containing (in mm): NaCl, 124; CaCl_2_, 2.5; MgCl_2_, 1.5; KCl, 1.6; KH_2_PO_4_, 1.2; NaHCO_3_, 24; glucose, 10; ascorbic acid, 2; pH 7.4, and bubbled with 95% O_2_–5% CO_2_. OGD was induced by replacing glucose with sucrose and by bubbling with 95% N_2_–5% CO_2_. In some experiments, psalmotoxin 1 (PcTx1, 100 nm, Pepta Nova, Sandhausen, Germany) was added in the medium or the extracellular solution (ACSF). At the concentration used this is expected to selectively block the calcium‐permeable ASIC1a channel (ASIC1a homomers and ASIC1a/2b heteromers), leaving calcium‐impermeable channels unaffected.

### Hippocampal pyramidal neuron culture and acidosis treatment

Cultured hippocampal neuronal (HPNs) were prepared from P1 Wistar rats using a previously described protocol with minor modifications (Hudmon *et al*. 2005). Cells were grown on glass coverslips or in 10 cm Petri dishes. Experiments were carried on cells 14–21 days *in vitro* (DIV). For experiments involving acidosis, after a quick wash with pH 7.4 solution, HPNs were challenged for 15 min with extracellular solution of different pH (pH 7.4 for controls, and pH 6.0 for acidosis) containing (in mm): NaCl, 145; KCl, 2.5; CaCl_2_, 1; MgCl_2_, 1; glucose, 10; Hepes, 10; Mes, 10. PcTx1 (20 nm) was added in the extracellular solution for some experiments.

### Electrophysiology on slices

AMPAR‐mediated fEPSPs and EPSCs were recorded as previously described (Quintana *et al*. 2006). Slices were placed in a homemade chamber and perfused with ACSF. For EPSPs, two recordings were made in parallel on the same slices with pipettes containing ACSF. The average of the two recordings was used for analysis. EPSP slope was defined as the maximum slope (d*V*/d*t*) of the initial portion of the response and was measured for all individual responses. Results were normalized to the average slope of fEPSPs recorded before OGD, and plotted as a function of time. fEPSPs were pure AMPAR‐mediated responses in our experimental conditions as NBQX (10 μm, Alexis Biochemicals, San Diego, CA, USA) suppressed the response (data not shown). EPSCs were measured in the presence of bicuculline (25 μm; Tocris Bioscience, Bristol, UK) or picrotoxin (100 μm; Tocris Bioscience), and d‐2‐amino‐5‐phosphonovalerate (d‐AP5, 50 μm; Alexis Biochemicals) to block respectively the GABA_A_ and NMDA receptors. Spermine tetrahydrochloride (100 μm, Tocris Bioscience) was added in the pipette solution (Kamboj *et al*. 1995). Average fEPSPs and EPSCs were obtained by aligning a minimum of five events (IGOR Pro, Wavemetrics Inc., Lake Oswego, OR, USA).

### Electrophysiology on HPNs

Twelve to twenty‐four hours after treatment, AMPAR currents were evoked in outside‐out patches by rapid application of 10 mm glutamate (100 ms pulses) as previously described (Soto *et al*. 2009), with 50 μm d‐AP5 added in the extracellular solution to block NMDA receptors. Average waveforms for illustration and analysis were generated after aligning a minimum of 20 events (NeuroMatic, IGOR Pro). Single‐channel conductance (γ, pS) was estimated using non‐stationary fluctuation analysis as previously described (Soto *et al*. 2009).

### Assessment of neuronal injury in OHSCs and HPNs

In experiments on OHSCs, we used propidium iodide (PI) uptake to assess cell injury. PI (5 μg ml^−1^) was added to the culture medium for 30 min to check slice viability before OGD or control treatment; OHSCs with significant staining were excluded from further analysis. Image acquisition was performed 12 and 24 h after OGD using an inverted fluorescence microscope (Axiovert, Zeiss), with a 2.5× magnification objective and a digital camera (Axiocam HRc, Zeiss). PI fluorescence was quantified in the CA1 region (arbitrary units) using ImageJ software (NIH) and the equivalent average signal in controls was subtracted from the value in OGD exposed slices. For each condition the signal of the different slices (*n* = 6–12/experiment) was averaged and a percentage of neuroprotection (NP) corresponding to the relative decrease in PI uptake induced by the blocker(s) compared with the OGD condition was calculated as follows:

NP %=[Avg PI Upt OGD −Avg PI Upt OGD + blocker ]Avg PI Upt OGD ×100

where *Avg* stands for average and *Upt* stands for Uptake.

The values obtained for the different experiments (*n* = 3 cultures) were averaged to obtain the final data. For ASIC1a KO OHSCs, experiments were repeated 4 times with 3–4 slices/experiment and image acquisition was performed at 2 and 24 h after OGD.

HPN cultures on coverslips were exposed to the different experimental solutions and stained 24 h post‐treatment with anti‐caspase‐3 (Asp175) (5A1E, Cell Signaling Technology, Danvers, MA, USA) and examined for nuclear fragmentation using DAPI staining (*n* = 6 coverslips per condition). Quantification was performed with ImageJ.

### Western blotting and cell surface biotinylation

We performed Western blotting (WB) on protein extracts from OHSCs as previously described with minor modifications (Quintana *et al*. 2006). OHSCs (3–4 independent cultures corresponding to 6–14 culture inserts per condition with 5–8 slices per insert) were collected and lysed in ice‐cold lysis buffer with Complete protease inhibitor mix (Roche). We used anti‐GluA1antibody (1:1000, Millipore, cat. no. 04–855), anti‐GluA2 antibody (1:1000, Millipore, cat. no. MAB397), anti‐actin antibody (1:5000, Sigma, cat. no. A5316), and anti‐NeuN (1:500, Millipore, cat. no. MAB377). For NeuN we quantified only the two major species at 46–48 kDa identified as Fox‐3 (Dent *et al*. 2010). GluA1, GluA2 and NeuN were quantified by normalizing to the respective actin bands. Immunoblots were visualized by ECL development (GE Healthcare Life Sciences) and using either films or the ChemiDoc MP System. The total levels of GluA1, GluA2 and NeuN were quantified by normalizing to the respective actin bands. For the quantification of the total GluA1/GluA2 ratio, to ensure consistency the same protein concentration was loaded; in addition each Western blot image was captured for identical lengths of time.

HPNs were grown in 10 cm Petri dishes and cell surface biotinylation was performed as previously described (Soto *et al*. 2009).

### Statistics

Data are expressed as means ± SEM. Statistical analyses were performed using GraphPad Prism (GraphPad Software Inc., La Jolla, CA, USA). We tested the distribution and the standard deviation of our datasets using the Shapiro‐Wilk normality test. We used unpaired Student's *t* test (parametric) or the Mann‐Whitney *U* test (non‐parametric) to compare pairs. To compare multiple experimental conditions, we used one‐way ANOVA (parametric) followed by Tukey's multiple comparison test or the Kruskal‐Wallis test (non‐parametric) followed by Dunn's multiple comparisons test.

## Results

### Anoxic LTP is ASIC1a dependent

To determine whether ASIC1a activation, during anoxia, influences AMPAR plasticity changes, we have tested whether a‐LTP is ASIC1a dependent. We measured AMPAR‐mediated field excitatory postsynaptic potentials (fEPSPs) in the stratum radiatum of the CA1 region of organotypic hippocampal slice cultures (OHSCs) prepared from wild‐type (WT) and ASIC1a knockout (KO) mice (Wemmie *et al*. 2002).

**Figure 1:**
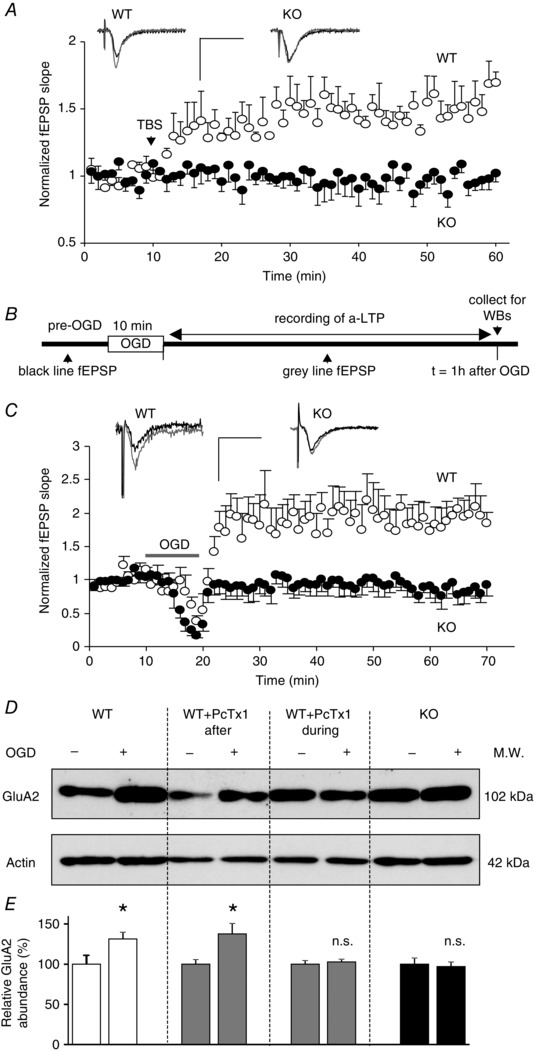
**Anoxic LTP relies on ASIC1a in OHSCs** *A*, theta‐burst stimulation (TBS) induced changes in the slope of AMPAR‐mediated fEPSPs measured in the CA1 stratum radiatum of OHSCs from WT (○, *n* = 4) and ASIC1a KO (●, *n* = 5) mice. Inset: representative baseline (black trace) and 30 min after TBS (grey trace) recordings; Scale bars: 25 ms and 1 mV. *B*, experimental protocol for a‐LTP experiments. *C*, changes of fEPSP slope induced by 10 min OGD in WT (○, *n* = 3) and ASIC1a KO (●, *n* = 4) OHSCs. The increase in slope observed in WT corresponds to a‐LTP. Inset: representative traces before (black trace) and 30 min after OGD (grey trace). Scale bars: 25 ms and 1 mV. *D*, representative Western blots showing changes in GluA2 subunit expression 1 h after OGD. OHSCs from WT or ASIC1a KO animals were exposed either to control (−) or to OGD (+) conditions. Some WT slices were treated with psalmotoxin 1 (PcTx1, 100 nm) during or after OGD; actin was used for normalization. *E*, relative GluA2 subunit abundance compared with controls expressed as a percentage (*n* = 6–14). Error bars, SEM; *P < 0.05; n.s., not significant.

To validate OHSCs as a model to study the role of ASIC1a in synaptic plasticity, we first tested the involvement of ASIC1a in theta‐burst stimulation (TBS)‐induced LTP. In WT slices, TBS (five trains at 5 Hz composed each of four pulses at 100 Hz, repeated three times at 10 s interval) was followed by a prolonged increase in fEPSP slope (average slope 45–50 min after TBS/average baseline = 1.52 ± 0.1, *n* = 4) which was absent in KO slices (slope ratio = 0.97 ± 0.1, *n* = 5) (Fig. 1 *A*), as previously described with acute slices (Wemmie *et al*. 2002). To induce a‐LTP, OHSCs were exposed for 10 min to conditions of oxygen glucose deprivation (OGD) (Fig. 1 *B*), a validated model of brain ischaemia. We have previously shown that this treatment induces a robust AMPAR‐mediated neurotransmission potentiation without increasing AMPAR rectification (Quintana *et al*. 2006). As expected from these observations, OGD treatment of WT slices induced a prolonged increase of fEPSP slope (average slope 45–50 min after OGD/average baseline = 1.97 ± 0.3, *n* = 3, Fig. 1 *C*). However, a‐LTP was absent in slices from ASIC1a KO animals (slope ratio = 0.85 ± 0.1, *n* = 4, Fig. 1 *C*).

Our previous data have also demonstrated that the maintenance of a‐LTP requires the synthesis of new GluA2 subunits (Quintana *et al*. 2006). Accordingly, we observed a significant increase in GluA2 expression 1 h after OGD in WT slices (GluA2_OGD_/GluA2_control_ = 1.31 ± 0.08, *n* = 8, *P* < 0.05, Fig. 1 *D* and *E*). Importantly, the increase in GluA2 expression was not apparent in ASIC1a KO slices (*n* = 7, Fig. 1 *D* and *E*) in agreement with the electrophysiology data. In addition, the ASIC1a‐specific inhibitor psalmotoxin 1 (PcTx1, 100 nm) blocked the upregulation of GluA2 in slices from WT mice if present during (*n* = 6) but not after (*n* = 14) OGD (Fig. 1 *D* and *E*). These results suggest that ASIC1a activation, during OGD, plays a role in the development and maintenance of ischaemia‐induced a‐LTP and the associated increase in AMPAR expression.

### OGD induced prevalence of CP‐AMPARs is ASIC1a dependent

**Figure 2:**
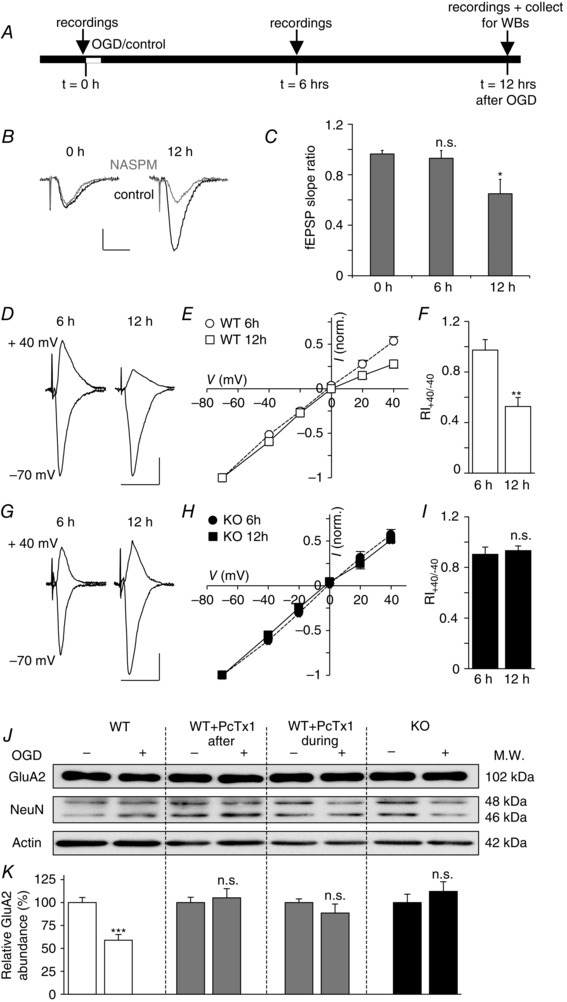
**OGD induced increase of CP‐AMPARs is ASIC1a dependent in slice cultures** *A*, experimental protocol. *B* and *C*, effect of NASPM (100 μm) on fEPSP slope before (0 h), 6 h after, and 12 h after OGD. Representative recordings (*B*), before (black traces) and after (grey traces) application of NASPM; scale bar, 25 ms and 0.5 mV. fEPSP slope ratio (*C*), after *vs*. before NASPM. *D–I*, changes in AMPAR‐mediated EPSCs recorded in CA1 neurons in cells derived from WT (*D–F*, *n* = 4–6) and ASIC1a KO (*G–I*, *n* = 4) mice. *D* and *G*, representative EPSC traces at +40 and −70 mV recorded 6 h (left hand side) and 12 h (right) after 10 min OGD treatment; scale bar, 25 ms and 25 pA. *E* and *H*, current–voltage plots at 6 h and 12 h after OGD. *F* and *I*, EPSC rectification index; RI_+40/−40_ = EPSC amplitude at +40 mV/−40 mV. *J* and *K*, changes in GluA2 subunit expression 12 h after OGD treatment. Cells from WT or ASIC1a KO animals were exposed either to control (−) or to OGD (+) conditions. Some WT slices were treated with psalmotoxin 1 (PcTx1, 100 nm) during or after OGD. *J*, representative Western blots, obtained for GluA2 subunits, NeuN and actin (for normalization). NeuN level is relatively stable. *K*, relative GluA2 abundance, compared with controls expressed as a percentage (*n* = 8–10). Error bars, SEM; *P < 0.05, **P < 0.005, ***P < 0.0005; n.s., not significant.

Ischaemia has been shown to induce a delayed increase in the expression of CP‐AMPARs in CA1 neurons due to a depression of GluA2 subunit expression in various animal models (Pellegrini‐Giampietro *et al*. 1992; Opitz *et al*. 2000; Tanaka *et al*. 2002; Noh *et al*. 2005). In our initial experiments, we tested if this could be reproduced *ex vivo* in OHSCs exposed to OGD. We observed that the CP‐AMPAR channel blocker NASPM (100 μm) did not affect the fEPSP slope measured before (0 h) or 6 h after OGD (ratio = slope after NASPM/before NASPM; ratio_0h_ = 0.97 ± 0.03, *n* = 7; ratio_6h_ = 0.93 ± 0.06, *n* = 3). However, by 12 h post‐OGD, NASPM significantly inhibited the response, confirming a delayed rise in synaptic CP‐AMPARs (ratio_12h_ = 0.65 ± 0.11, *n* = 4, *P* < 0.05), similar to that described *in vivo* (Noh *et al*. 2005) (Fig. 2 *A–C*).

To investigate the potential role of ASIC1a in this process, we examined evoked excitatory postsynaptic currents (EPSCs) in whole‐cell recordings from CA1 neurons in OHSCs from WT and ASIC1a KO mice 6 and 12 h after OGD (Fig. 2 *D–I*). The presence of EPSCs mediated by CP‐AMPARs was readily identified from their inwardly rectifying current–voltage (*I–V*) relationships and low rectification index (RI; amplitude EPSC at +40 mV/amplitude EPSC at −40 mV) when intracellular polyamine (spermine, 100 μm) was included in the patch pipette (Kamboj *et al*. 1995).

In WT neurons there was an increase in inward rectification of EPSCs, and a significant drop in their RI at 12 *vs*. 6 h post OGD (RI_6h_ = 0.97 ± 0.08, RI_12h_ = 0.53 ± 0.07, *P* < 0.005, *n* = 4–6, Fig. 2 *D–F*), indicating a time‐dependent rise in the proportion of synaptic CP‐AMPARs. As previously noted *in vivo*, this change was associated with a clear downregulation of the GluA2 subunit (GluA2_OGD_/ GluA2_control_ = 0.59 ± 0.06, *n* = 8, *P* < 0.0005, Fig. 2 *J* and *K*). The neuron specific protein NeuN decreased significantly 12 h after OGD reflecting neuron death (NeuN_OGD_/NeuN_control_ = 0.8 ± 0.06, *P* < 0.05, *n* = 8, Fig. 2 *J*). However, we found no positive correlation between the level of GluA2 and NeuN (*R* ^2^ = 0.53) suggesting that neuron death was not the primary cause of the reduction in the GluA2 level. The absence of ASIC1a channels or their block by PcTx1, during or after OGD, suppressed these AMPAR changes (Fig. 2 *G–K*) suggesting that ASIC1a activation, during and after OGD, is necessary for the late increase in GluA2‐lacking CP‐AMPARs.

**Figure 3:**
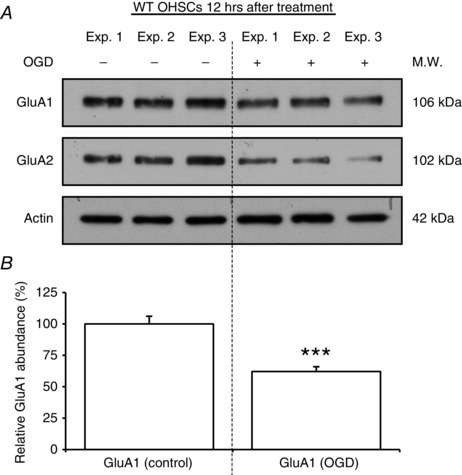
**GluA1 subunit is downregulated in OHSCs exposed to OGD** *A* and *B*, changes in GluA1 subunit expression 12 h after OGD treatment. OHSCs from WT animals were exposed either to control (−) or to OGD (+) conditions. *A*, Western blots, obtained for GluA1 subunits, GluA2 subunits and actin (for normalization) in three representative experiments (Exp. 1–3). *B*, relative GluA1 abundance, compared with controls expressed in percentage (*n* = 8). GluA1 subunit abundance decreases in a similar way to GluA2 in total protein extracts of OHSCs. Error bars, SEM; ***P < 0.0005.

Despite convergent evidence indicating an increase in CP‐AMPARs, we found that GluA1 appeared to be similarly downregulated in WT slices exposed to OGD (GluA1_OGD_/GluA1_control_ = 0.62 ± 0.04, *n* = 8, *P* < 0.0005, Fig. 3). And like GluA2, in all other conditions it was not significantly changed (*P* > 0.5). Thus, we found no change following OGD in ASIC1a KO slices (GluA1_OGD_/GluA1_control_ = 1.12 ± 0.23, *n* = 9), or in WT mice when OGD was followed by PcTx1 (GluA1_OGD_/GluA1_control_ = 0.82 ± 0.05, *n* = 10), or accompanied by PcTx1 (GluA1_OGD_/ GluA1_control_ = 0.79 ± 0.11, *n* = 8). To clarify this point and assess the mechanisms underlying the delayed changes in AMPARs, we next performed experiments on purified cultures of hippocampal pyramidal neurons (HPNs).

### H^+^ activation of ASIC1a is sufficient to induce an increase in CP‐AMPARs

**Figure 4:**
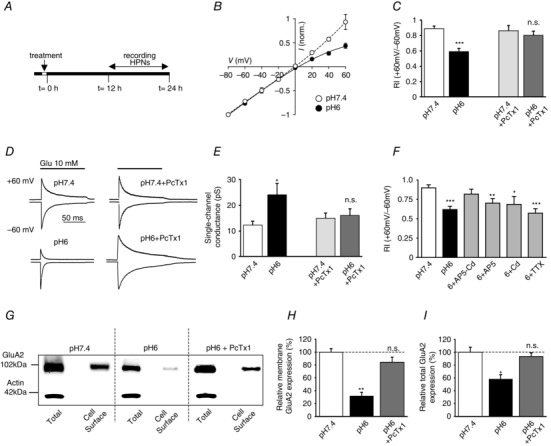
**Acidosis induces an ASIC1a‐dependent switch in AMPAR composition in cultured neurons** *A*, experimental protocol. *B*, *I–V* plots of AMPAR currents measured in outside‐out patches from hippocampal pyramidal neurons (HPNs) exposed to pH 7.4 (control, ○, *n* = 3) or pH 6.0 (acidosis, ●, *n* = 7). *C*, AMPAR current rectification index, in control cells and following pH 6.0 treatment; effect of pH 6.0 on RI is blocked by psalmotoxin 1, (PcTx1, 20 nm) (RI_+60/−60_, *n* = 11–26). *D*, typical current traces at +60 and −60 mV. For ease of comparison, currents at −60 mV are scaled to the same peak amplitude. Upper traces: HPNs exposed to pH 7.4 (left) or pH 7.4 + PcTx1 (right). Lower traces: HPNs exposed to pH 6.0 (left) or pH 6.0 + PcTx1 (right). *E*, AMPAR single‐channel conductance (*n* = 7–16). *F*, bar graph comparing the effects of pH 6.0 treatment in the presence of d‐AP5 (50 μm), cadmium (200 μm) and TTX (1 μm) on AMPAR rectification index. *G*, Western blots of total and cell surface expression of GluA2 in HPNs 12 h after treatment, characterized by cell surface biotinylation followed by Western blotting (WB) (*n* = 8). *H* and *I*, pooled data from pH 7.4 and pH 6.0 treated hippocampal cultures assayed for total and surface (membrane) GluA2. Error bars, SEM; *P < 0.05, **P < 0.005, ***P < 0.0005; n.s., not significant.

There is evidence to suggest that neuronal ASIC1a can be activated by vesicular protons co‐released with glutamate (Mari *et al*. 2010; Du *et al*. 2014; Kreple *et al*. 2014) and by the acidosis that follows stroke (Pignataro *et al*. 2007). To assess if proton activation of ASIC1a channels in hippocampal pyramidal neurons (HPNs) will trigger the AMPAR switch, we measured the *I–V* relationship of currents obtained in response to rapid applications of glutamate (10 mm, 100 ms duration, in the presence of d‐AP5) to outside‐out membrane patches from cultured HPNs. Our hippocampal cultures contained predominantly neurons (see Fig. 6 *D*) and allowed rapid change of the cellular environment. These cells were exposed to either an increased level of protons (pH 6.0 or 6.5) or normal physiological pH (7.4) for 15 min (Fig. 4 *A*), and the AMPAR currents were examined 12 to 24 h after this treatment (Fig. 4 *B–F*).

Compared with control cells, neurons exposed to pH 6.0 displayed inwardly rectifying *I–V* plots indicative of the presence of CP‐AMPARs (Fig. 4 *B*). The rectification index of currents in patches (RI = current amplitude at +60 mV/−60 mV) was reduced (from RI_pH7.4_ = 0.89 ± 0.03 to RI_pH6_ = 0.59 ± 0.04, *P* < 0.0001, *n* = 23–26, Fig. 4 *C* and *D*), and the single‐channel conductance (γ), estimated from non‐stationary fluctuation analysis (Soto *et al*. 2009), was roughly doubled (γ_pH7.4_ = 12.33 ± 1.38 pS, γ_pH6_ = 24.14 ± 4.36 pS, *P* < 0.05, *n* = 15–17, Fig. 4 *E*). Strong inward rectification and a high single‐channel conductance are both indicative of CP‐AMPARs. HPNs exposed to pH 6.5 did not exhibit significant changes in the rectification index or single‐channel conductance of their AMPAR‐mediated currents (RI_pH6.5_ = 0.89 ± 0.11, γ_pH6.5_ = 14.61 ± 3.03; *n* = 6).

Consistent with the view that CP‐AMPAR expression was increased 12 h after pH 6.0 acidosis, we found a significant decrease in GluA2 both at the cell surface and in total protein expression (sGluA2_pH6.0_/sGluA2_pH7.4_ = 34.26 ± 7.2 %, *P* < 0.005 and tGluA2_pH6.0_/tGluA2_pH7.4_ =  59.8 ± 10 %, *P* < 0.05, *n* = 8, Fig. 4 *H–I* ). Application of PcTx1 (20 nm) during pH 6.0 treatment was able to suppress these changes, and changes in rectification and single‐channel conductance (RI_pH6+PcTx1_ = 0.80 ± 0.06, *n* = 16, γ_pH6+PcTx1_ = 16.11 ± 2.45 pS, *n* = 10, Fig. 4 *C–E*).

ASIC stimulation depolarizes the membrane potential of hippocampal neurons, modulating voltage‐gated ion channel and NMDAR activation. To test if this influenced the ASIC‐induced switch in AMPAR composition, we included selective ion channel blockers in the pH 6.0 solution. The block of voltage‐gated calcium channels (VGCCs) by cadmium (Cd 200 μm) (RI_pH6+Cd_ = 0.69 ± 0.10, γ_pH6+Cd_ = 21.91 ± 4.72 pS, *n* = 14, Fig. 4 *F*) and of NMDARs by d‐AP5 (50 μm) (RI_pH6+AP5_ = 0.70 ± 0.06, γ_pH6+AP5_ = 16.99 ± 2.68 pS, *n* = 16, Fig. 4 *F*) partially suppressed the change in AMPAR properties. Inhibiting both VGCCs and NMDARs almost completely blocked the AMPAR subunit switch induced by ASIC1a activation (RI_pH6+Cd+AP5_ = 0.85 ± 0.06, *n* = 16, γ_pH6+Cd+AP5_ = 14.72 ± 1.82 pS, *n* = 13, Fig. 4 *F*). However, blocking sodium channels with tetrodotoxin (TTX 1 μm) did not inhibit the change (RI_pH6+TTX_ = 0.57 ± 0.06, γ_pH6+TTX_ = 22.04 ± 3.10 pS, *n* = 16, Fig. 4 *F*). These results suggest that modulation of Ca^2+^‐permeable NMDARs and VGCCs by ASIC1a activation is critical in triggering the observed AMPAR plasticity, suggesting Ca^2+^ influx is required for the loss of GluA2 expression detected following acidification.

**Figure 5:**
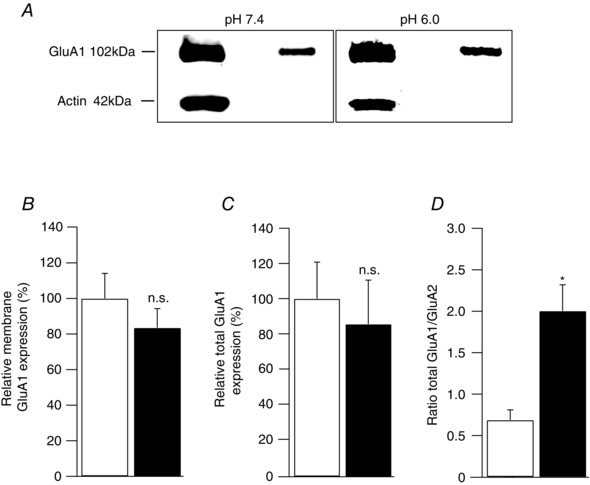
**Acidification does not alter GluA1 expression in pyramidal neurons** *A*, Western blot analysis of total and cell surface GluA1 from cultured hippocampal pyramidal neurons (HPNs) 12 h after exposure to either pH 7.4 or pH 6.0. *B* and *C*, pooled data from 5 independent cell surface biotinylation experiments demonstrating no change in cell surface expression of GluA1 following acidification (*P* > 0.05, *n* = 5). *D*, Western blot analysis of the ratio of total protein expression level of GluA1 to the total of GluA2 (GluA1/GluA2) following acidification. This ratio increased significantly (0.70 ± 0.12 to 2.0 ± 0.32; *P* < 0.05, *n* = 5).

We extended these findings by examining the effects of acidosis on the expression levels of GluA1 (Fig. 5), and found no alteration in either the total (Fig. 5 *C*) or cell surface GluA1 (Fig. 5 *B*). Consequently, proton activation of ASIC1a channels increased the ratio of total GluA1/total GluA2 from 0.70 ± 0.12 to 2.0 ± 0.32  (*P* < 0.05, *n* = 5, Fig. 5 *D*), suggesting the observed switch from Ca^2+^‐impermeable to CP‐AMPARs in HPNs can be ascribed to a relative decrease in GluA2.

### Combined inhibition of ASIC1a and CP‐AMPARs does not enhance neuroprotection

**Figure 6:**
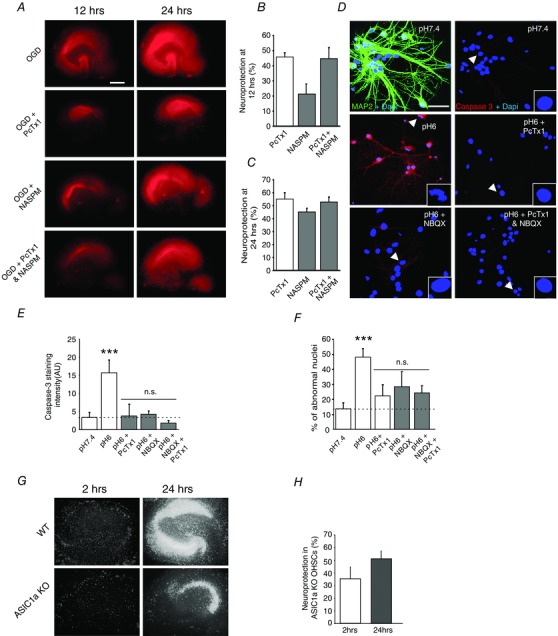
**Combining ASIC1a and CP‐AMPAR inhibitors does not provide further neuroprotection against ischaemia or acidosis** *A*, evaluation of OGD‐induced degeneration in organotypic cultures with propidium iodide (PI) uptake quantification. Representative pictures of PI uptake in slices at 12 and 24 h after OGD. Scale bar = 250 μm. WT slices exposed to OGD were treated for the first 6 h with either saline (control), PcTx1 (100 nm), NASPM (100 μm) or both inhibitors together. *B* and *C*, quantification of the neuroprotection by ion channel inhibitors in the CA1 region at 12 and 24 h after OGD. The percentage of neuroprotection was calculated as detailed in Methods (*n* = 3 cultures, number of slices/condition = 6–12). *D*, confocal images of hippocampal pyramidal cells 24 h after a 15 min treatment with pH 7.4, pH 6.0, pH6.0 + PcTx1 (20 nm, during pH 6.0), pH 6.0 + NBQX (10 μm, after pH 6.0), or pH 6.0 + PcTx1 and NBQX together. Top left, double staining for MAP2 (green) and DAPI (blue) confirms the purity of HPN cultures. Other pictures, neuronal injury and death in HPN cultures assessed with caspase‐3 cleaved form staining (red) and the percentage of abnormal nuclei (stained in blue with DAPI) as markers. Scale bar = 25 μm. Typical nuclei are indicated by an arrowhead and represented at high magnification in the right bottom corner. *E* and *F*, quantification of the cleaved caspase‐3 signal, and of the percentage of abnormal nuclei. *G*, representative images of propidium iodide uptake in hippocampal slices from WT and ASIC1a knockout at 2 and 24 h after OGD. *H*, pooled data showing the protective effect of ASIC1a knockout on OGD mediated cell death (*n* = 4). Error bars, SEM; *P < 0.05, **P < 0.005, ***P < 0.0005 *vs*. pH 7.4; n.s., not significant.

Block of either ASIC1a channels or CP‐AMPARs is known to reduce drastically cell death following stroke (Xiong *et al*. 2004; Noh *et al*. 2005; Pignataro *et al*. 2007). We therefore considered whether the combined use of ASIC1a and AMPAR blockers produced an additive effect in sparing neurons from damage (Fig. 6). To estimate the degree of neuron death following OGD in OHSCs, we measured propidium iodide (PI) uptake at 12 and 24 h after OGD (Fig. 6 *A*).

As previously described (Ahlgren *et al*. 2011), the OGD induced injury of CA1 cells was already evident within 12 h and had increased dramatically by 24 h. We then estimated the percentage of neuroprotection (see Methods) provided by inclusion in the bathing medium of PcTx1 (100 nm) during the first 6 h after OGD, or by NASPM (100 μm) after the first 6 h, when applied either alone or successively (Fig. 6 *A*). The presence of PcTx1 produced a clear decrease in cell death at 12 and 24 h (NP_12h_ = 45.71 ± 2.68%, NP_24h_ = 55.03 ± 5.82%, *n* = 3 experiments with 6–12 slices/experiment; Fig. 6 *B* and *C*). By contrast, while NASPM provide some protection at 12 h, it was less effective than PcTx1 (NP_12h_ = 21.16 ± 6.78%, Fig. 6 *B* and *C*). By 24 h, it appeared to be roughly as effective as PcTx1 (NP_24h_ = 45.11 ± 2.95%). This is likely to reflect the delayed increase in CP‐AMPARs. However, when NASPM and PcTx1 were applied together they did not appear to provide more protection than PcTx1 alone (NP_12h_ = 44.46 ± 7.58%, NP_24h_ = 52.56 ± 3.94%) (Fig. 6 *B* and *C*). This is consistent with our finding that the presence of PcTx1 after OGD effectively suppressed the increased expression in CP‐AMPARs.

To confirm the role of ASIC1a in OGD induced cell death, we examined PI uptake in CA1 from WT (control) and ASIC1a KO hippocampal slices following OGD (Fig. 6 *G and H*).There was a clear decrease in cell death at 2 and 24 h after OGD treatment in the ASIC1a KO tissue, when compared with WT (NP_2h_ = 35.41 ± 9.33 % and NP_24h_ = 51.00 ± 6.39 %; *n* = 4 experiments, Fig. 6 *H*). These data indicate that ASIC1a KO affords some neuroprotection, in agreement with our hypothesis.

To examine this issue further, we counted nuclear abnormalities (nuclei stained with DAPI) and measured cleaved caspase‐3 staining in cultured HPNs. Neuron injury was assessed 24 h after acidosis (pH 6.0 for 15 min) (Fig. 6 *D*, *n* = 6 coverslips per condition). Neurons exposed to pH 6.0 exhibited an increased level of cleaved caspase‐3 expression (*P* < 0.005) and an increased density of abnormal nuclei (*P* < 0.005) (Fig. 6 *E* and *F*) compared with controls (pH 7.4). When PcTx1 (20 nm) was included during exposure to pH 6.0, neuronal damage was significantly reduced (*P* < 0.005 for both markers, Fig. 6 *E* and *F*). Furthermore, inclusion of the potent AMPAR blocker NBQX (10 μm) after exposure to pH 6.0 also provided protection from cell death (*P* < 0.05 for nuclei, and *P* < 0.005 for caspase‐3, Fig. 6 *E* and *F*). However, blocking both ASIC1a and CP‐AMPARs together did not bring significant further protection compared with block of ASIC1a channels alone (*P* > 0.05, *n* = 6 coverslips per condition, Fig. 6 *E* and *F*). These results are consistent with the view that activation of ASIC1a channels during OGD is critical in driving hippocampal neuron damage.

## Discussion

Our experiments have established that ASIC1a channels play a key role in AMPAR plasticity in conditions of ischaemia or acidosis in HPNs. We find that ASIC1a activation is critical for development and maintenance of a‐LTP in CA1 neurons. Furthermore, activation of ASIC1a channels is necessary and sufficient to trigger the deleterious increase in CP‐AMPARs that occurs following ischaemia or acidosis. Consistent with these results, we observed that inhibiting ASIC1a circumvented the potential neuroprotective benefit afforded by the use of CP‐AMPAR blockers.

We demonstrated that ASIC1a activation is necessary for the pathological form of synaptic plasticity, a‐LTP, and confirmed its role in activity‐dependent LTP in CA1 cells (Wemmie *et al*. 2002). These results are consistent with previous studies suggesting these two forms of plasticity share a number of common features including NMDAR dependence (Hsu & Huang, 1997; Quintana *et al*. 2006). Therefore, ASIC1a might contribute to a‐LTP by facilitating activation of the NMDARs as suggested for activity‐dependent LTP (Wemmie *et al*. 2002). It is also of note that ASIC1a channels are permeable to both Na^+^ and Ca^2+^. Thus, their activation would be expected to increase intracellular Ca^2+^, directly or indirectly, and potentially contribute to LTP induced by activity or anoxia.

While the activity‐dependent form of LTP is thought to involve the transient incorporation of GluA2‐lacking CP‐AMPARs (Plant *et al*. 2006; but see Adesnik & Nicoll, 2007), it is notable that in our OGD model, we found a transient increase in total GluA2 during the first hour following OGD. Furthermore, the fact that we detected no rapid change in rectification during the first hour following OGD (see also Quintana *et al*. 2006), but only at later times, contrasts with some earlier studies on acute slices which found an early increase in rectification after OGD (Dixon *et al*. 2009; Dias *et al*. 2013). These various observations suggest that the different slice preparations used by different groups might influence the speed of insertion or the synaptic localization of CP‐AMPARs following OGD. Another critical factor may be the OGD protocol itself. Dixon *et al*. (2009) performed a 30 min OGD, compared with our 10 min OGD. Dias *et al*. (2013) used 10 min OGD but in the presence of 3 mm glucose and at room temperature. All of these factors (duration of OGD, presence of glucose, and temperature) could influence the speed of post‐ischaemic AMPAR plasticity and affect neuronal fate after ischaemia.

The delayed decrease in total GluA2 we detected in organotypic slices is in keeping with the general observation that inward rectification of synaptic AMPARs increased. However, it differs from previous work on cultured hippocampal neurons by Liu *et al*. (2006) and Fernandes *et al*. (2014). These two studies found no change in total GluA2 protein abundance at various time points up to 24 h post‐OGD. The fact that we also detected a downregulation of GluA1 was unexpected, in view of the electrophysiology data showing that EPSCs were more rectifying at 12 h in WT slices exposed to OGD. The reason for this is unclear, but the fact that increased targeting of CP‐AMPAR (GluA2‐lacking) subtypes to synaptic sites occurred following OGD suggests either it also involves other AMPAR subunits, or that even in conditions of reduced GluA1, sufficient CP‐AMPARs are targeted to synapses to cause a functional change in EPSC properties.

In addition, we calculated the ratio of total GluA1 expression to total GluA2 following acidification of pure HPN cultures. While we found evidence for a decrease in total GluA2 but not in GluA1 following ASIC1a activation, we identified a significant increase in the GluA1/GluA2 ratio. This increased ratio resembles the relative decrease in GluA2 expression observed in CA1 following OGD (Pellegrini‐Giampietro *et al*. 1992). Overall, our study using two different models underlines that ASIC1a activation by acidosis is necessary and sufficient to induce the increased CP‐AMPAR expression that is associated with ischaemia and excitotoxicity *in vivo* (Pellegrini‐Giampietro *et al*. 1992; Tanaka *et al*. 2002; Noh *et al*. 2005).

Interestingly, a recent study has demonstrated an opposite role for ASIC1a in the nucleus accumbens of the ventral striatum. In these neurons, ASIC1a deletion increases CP‐AMPAR prevalence (Kreple *et al*. 2014). ASIC1a‐mediated calcium influx has been shown to induce the Ca^2+^‐dependent translocation of the nuclear factor of activated T cells (NFATc) (Li *et al*. 2013). NFATc promotes GluA2 expression in striatal neurons (Groth *et al*. 2008) but represses it in hippocampal neurons (P. G. Mermelstein, personal communication). Therefore, ASIC1a could regulate GluA2 expression and CP‐AMPAR prevalence in a tissue specific way through a direct activation of the NFATc pathway. Based on our observations, ASIC1a could also activate NFATc, decreasing GluA2 expression in HPNs, by increasing Ca^2+^‐permeable NMDAR‐ and VGCC‐mediated currents. Our results highlight the need for further investigation to determine if ASIC1a may modulate CP‐AMPAR expression in other CNS structures and in diseases that involve both CP‐AMPARs and ASIC1a channels (such as spinal cord injury and epilepsy) (Weiss, 2011; Wemmie *et al*. 2013).

We also observed that blocking both ASIC1a and CP‐AMPARs appears to be neither additive nor synergistic, in terms of neuroprotection, as ASIC1a inhibitor suppresses expression of CP‐AMPARs. This may be useful in refining future combinatorial therapeutic strategies for stroke (Nakka *et al*. 2008). The present findings are consistent with the view that acidosis may contribute to neuronal damage not only as a result of Ca^2+^ influx through calcium‐permeable ASIC1a channels (Xiong *et al*. 2004; Mari *et al*. 2010), but also as result of delayed expression of GluA2 lacking CP‐AMPARs (Noh *et al*. 2005). While over‐activation of NMDA receptors has been shown to contribute to ASIC1a‐dependent damage that occurs during ischaemia (Gao *et al*. 2005), the increased level of protons is itself known to alter markedly a number of other important aspects of synaptic transmission in divergent ways (Chesler, 2003). Thus, an increase in protons inhibits NMDAR channel activity by trapping channels in a non‐conducting state (Traynelis & Cull‐Candy, 1990; Banke *et al*. 2005). Moreover, mediators of ischaemia, such as arachidonic acid, have been shown to modulate differentially ASICs and ionotropic glutamate receptors (Miller *et al*. 1992; Kovalchuk *et al*. 1994; Allen & Attwell, 2002). Therefore, our results provide an insight into the complex and still poorly understood interactions between acido‐ and excitotoxicity.

In conclusion, we propose that ASIC1a activation can drive certain forms of CP‐AMPAR plasticity, and that inhibiting ASIC1a affords neuroprotection.

## Additional information

### Competing interests

The authors declare no competing financial interests.

### Author contributions

O.P., P.Q., D.S., M.Z. and S.G.C.‐C. designed the research; P.Q., D.S., O.P. and M.Z. performed the research and analysed the data; D.M., R.C. and S.K. provided materials and feedback; and O.P. and S.G.C.‐C. wrote the paper with input from D.S. and M.Z. All authors approved the final version of the manuscript for publication.

### Funding

This work was supported by the EMBO fellowship programme (ALTF 1017–2006 to O.P.), the Swiss National Science Foundation (PALAB–115678 to O.P., grant 31003A 135735/1 to R.C., grant 31–56852.99 to D.M., grant 3100A0‐105262 to S.K.), Programme Grants from the Medical Research Council (MR/J002976/1 for S.G.C.‐C. and Mark Farrant), and the Wellcome Trust (086185/Z/08/Z to S.G.C.‐C. and MF). J.N.W. was supported by the MRC and Wellcome Trust.
